# A Novel Data-driven Approach to Examine Children’s Movements and Social Behaviour in Schoolyard Environments

**DOI:** 10.3390/children9081177

**Published:** 2022-08-05

**Authors:** Maedeh Nasri, Yung-Ting Tsou, Alexander Koutamanis, Mitra Baratchi, Sarah Giest, Dennis Reidsma, Carolien Rieffe

**Affiliations:** 1 Unit of Developmental and Educational Psychology, Institute of Psychology, Leiden University, 2300 RB Leiden, The Netherlands; y.tsou@fsw.leidenuniv.nl (Y.-T.T.); crieffe@fsw.leidenuniv.nl (C.R.); 2 Leiden-Delft-Erasmus Centre for BOLD Cities, Leiden University, 2300 RA Leiden, The Netherlands; 3 Department of Computer Science, Faculty of Science, Vrije Universiteit Amsterdam, 1081 HV Amsterdam, The Netherlands; 4 Faculty of Architecture & the Built Environment, Delft University of Technology, 2628 BL Delft, The Netherlands; a.koutamanis@tudelft.nl; 5 Leiden Institute of Advanced Computer Science, Leiden University, 2300 RA Leiden, The Netherlands; m.baratchi@liacs.leidenuniv.nl; 6 Institute of Public Administration, Faculty of Governance and Global Affairs, Leiden University, 2511 DC The Hague, The Netherlands; s.n.giest@fgga.leidenuniv.nl; 7 Human Media Interaction Research Group, Faculty of Electrical Engineering, Mathematics and Computer Science, University of Twente, 7522 NB Enschede, The Netherlands; d.reidsma@utwente.nl; 8 Department of Psychology and Human Development, Institute of Education, University College London, London WC1H 0AA, UK

**Keywords:** children, affordances, social behaviour, schoolyard design

## Abstract

Social participation in schoolyards is crucial for children’s development. Yet, schoolyard environments contain features that can hinder children’s social participation. In this paper, we empirically examine schoolyards to identify existing obstacles. Traditionally, this type of study requires huge amounts of detailed information about children in a given environment. Collecting such data is exceedingly difficult and expensive. In this study, we present a novel sensor data-driven approach for gathering this information and examining the effect of schoolyard environments on children's behaviours in light of schoolyard affordances and individual effectivities. Sensor data is collected from 150 children at two primary schools, using location trackers, proximity tags, and Multi-Motion receivers to measure locations, face-to-face contacts, and activities. Results show strong potential for this data-driven approach, as it allows collecting data from individuals and their interactions with schoolyard environments, examining the triad of physical, social, and cultural affordances in schoolyards, and identifying factors that significantly impact children’s behaviours. Based on this approach, we further obtain better knowledge on the impact of these factors and identify limitations in schoolyard designs, which can inform schools, designers, and policymakers about current problems and practical solutions.

## 1. Introduction

Children spend a considerable amount of time in schoolyards, engaging in loosely structured activities under relatively mild supervision. The schoolyard environment, therefore, presents unique opportunities for children to play and develop their physical and social skills. Unfortunately, schoolyards may also pose unexpected obstacles that limit social play in various ways. For example, poor acoustics might hinder children who face barriers to communication, including children with hearing loss or autistic children. Examining children's behaviour in the context of the particular environment where it occurs could aid in identifying existing limitations and possibilities for schoolyard design. It could also inform the development of methods for improving schoolyards, and make it possible to tailor designs for sensitivity to differences in children’s needs, desires, and capacities. This, in turn, could help maximise opportunities for social learning for all children, including those who belong to vulnerable populations.

However, making this picture clearer requires a huge amount of data: precise information gathered over time, pertaining to not only different aspects of children’s behaviour, but also to the specific environment in which the behaviour occurs. This study, therefore, presents a sensor data-driven approach for gathering the information necessary for examining the effects of the schoolyard environment on children's movements and social behaviours. 

### 1.1. Affordances and Effectivities in Physical, Social, and Cultural Environments in Schoolyards

When children are in a schoolyard, they are constantly confronted with at least three layers of the schoolyard environment: the physical/built environment (physical layout and features) [1,2], the social environment (people to interact with) [3,4], and the cultural environment (rules and constraints set by schools) [5,6,7]. All three layers present different affordances to the children (see Box 1). Affordances are the actionable properties an environment presents to a child (e.g., a sand-pit affords building a sandcastle), in relation to the children’s individual desires, needs, and capacities [8]. That is, an environment’s affordances are relative to specific actions. For example, a sand-pit that is empty (without any sand) makes no difference in affording opportunities to a child who wants to be running around. Yet, it stops those who want to build a sandcastle from doing so. Certain interactions require an appropriate setting: a quiet, secluded corner for confidential talks, or a wide-open area for a large game involving physical activity. Certain settings stimulate certain activities and behaviours, as one can observe around any piece of schoolyard equipment; and certain activities are subject to school rules and conditions, e.g., football or cycling may be permitted only at specific places and in particular times.

Several studies incorporated a perspective on affordances to focus on interactions of children in general with their physical, social, and/or cultural environment. Many affordance studies concerning children, schoolyards, and schools depart from Heft's categorizations [8]. Heft distinguished between ten types of outdoor environments, such as "flat, relatively smooth surfaces" (which may afford walking, running, cycling, skating, skateboarding) or "attached objects" (which may afford sitting-on, jumping-on/over/down-from), and further extends affordances to include social and emotional behaviours [9,10]. Physical affordances are mostly studied in children-oriented research, including studies on how different environments (e.g., home, school, sport, leisure, neighbourhood, outdoor play) either promote or hinder various motor activities [11,12,13], and how physical, social, and cultural affordances influence physical activity levels in schoolyards [14,15]. 

Yet, when studying how schoolyard environments afford opportunities for children to play, it is important to consider all three layers of the environment: physical, social, and cultural. These are closely intertwined, and ignoring any layer could bias any data analyses and interpretations. For example, if a play area is too small, children who come late to the game may be excluded simply because there is no room for them. Such an outcome due to capacity issues may not necessarily reflect social exclusion. However this example illustrates restrictions imposed by the design and operation of a schoolyard. Moreover, if the social environment is not taken into account when considering the physical and cultural characteristics of a particular schoolyard, then schools, designers, and policymakers could be kept unaware of the limitations and possibilities of that schoolyard. Consequently, opportunities for improvement could be missed. 

Of particular interest are vulnerable children (e.g., children with a clinical diagnosis or disability) who might have different desires, needs, and capacities (“effectivities”) in their use of space as compared to other children in the same environment. For example, autistic children may be sensitive to certain ambient triggers (sounds, light, or touch) or avoid being in crowded areas [16,17,18]. They often prefer repetitive games with predictable results, such as spinning, twirling, and illuminating [19,20] and fixed routines, with clear instructions and rules to follow [21]. Autistic children can also find initiating or maintaining social contacts with other peers quite challenging [22]. Children with attention deficit hyperactivity disorder (ADHD) are observed to often change activities during breaktime, and many ADHD children have difficulties sustaining interactions with peers [23,24,25]. Thus, for vulnerable children, it may be especially critical to unravel the relationship between their individual interactions and their environment. 

By identifying affordances, we can be explicit and transparent in two critical aspects that are especially relevant to the inclusion of vulnerable children in schoolyards. First, working through a lens of affordances makes an explicit definition about vulnerable children’s capacity, so as to know what they expect, want or can do. This, consequently, facilitates awareness of special needs. This is notable because although special needs are usually considered in the teaching activities in the classroom, they may be ignored in the design and use of the schoolyard. Second, taking affordances into consideration clarifies the influences of the physical environment on children’s activities. Consequently, making affordances for vulnerable children explicit can help identify existing limitations, and develop methods for the analysis and evaluation of school environments. 

Taken together, we see a need to understand individual interactions at the micro-level of peripersonal space, while still taking into account the physical, social and cultural layers of the children’s direct environment, and the challenges they may pose for vulnerable children. To the best of our knowledge, there are no prior studies that examine how the environmental triad of physical, social, and cultural affordances interact with children’s behaviours and movements in the schoolyard. Moreover, in available affordance studies, vulnerable children have never been considered. While a large body of literature has reported on vulnerable children's physical activity levels, forms of play, and social connectedness in the schoolyard [26,27,28,29], outcomes that were reported were not linked to any environmental factors.

Box 1 **Affordances in schoolyards**

For the purposes of our research, we distinguish between three levels of affordances:
  - *Physical affordances*: what the physical layout and features of the schoolyard afford to children and their activities. These are critical for many vulnerable children, to the extent that they may even exclude themselves from what takes place in the schoolyard. For example, what most humans tolerate as mild background noise can be insufferable to children with cochlear implants, who, consequently, tend to refrain from entering schoolyard areas where exposed to such noise [30,31].
  - *Social affordances*: these refer to two complementary matters:
    - *What features in the schoolyard afford social interaction*, i.e., social interactions in our case, should be accommodated and facilitated by the environment. For example, having a chat with a classmate requires some sitting furniture in a quiet part of the schoolyard. This involves not only the need for a suitable environment for social interactions but also the features in the environment that stimulate social interactions (such as the presence of a seesaw, which invites play with another child).
    - *How the presence of others adds to or detracts from the affordances of the physical environment*. For example, if a swing is already occupied by another person, then the child is unable to sit on it. However, a new affordance becomes available: for example, pushing the person sitting on the swing.
  - *Cultural affordances*: free play and schoolyard use are normally subject to constraints, where, for example, some intensive or hazardous activities (such as football or cycling) are allowed only in certain parts of the schoolyard, or for a specific period of time.

### 1.2. Present Study

How do we identify a schoolyard’s affordances in relation to the effectivities of the children who use it? Most of the previous affordance studies relied on qualitative data, such as observations and self-reports. Although informative, these methods might not examine different interconnected aspects in a cost-effective way, nor give the detailed level of information necessary to draw reliable conclusions. Yet these objectives might be achieved by using the newly available sensor technologies. Sensor data promise comprehensive coverage of what takes place in a schoolyard at a low cost. They make continuous, objective monitoring of activities and interactions feasible. They provide reliable reports on schoolyard performance, and enable schools to identify problems as soon as they emerge. Some recent studies applied Global Positioning System (GPS) trackers and accelerometers. This new data-driven approach has been used, for example, to examine physical activity levels [32], and to compare active outdoor play in schoolyards and in natural environments, taking into account personal characteristics (e.g., age) as well as the physical and social environment [33,34,35,36]. 

The main challenge is to collect such precise information from different layers and unravel the complex relationship between environmental affordances in schoolyards and children’s effectivities. To deal with this, we designed a data-driven approach for collecting data that would feature enough detail and precision to inform us about relations among three different environmental layers (physical, social and cultural) and the children’s role in these. We, then, examined the extent to which children’s movements and social behaviours were affected by the physical, social, and cultural affordances of a schoolyard by identifying three successive aims. 

First, we aimed to develop a novel sensor data-driven approach by integrating unobtrusive data collection. This technology included GPS loggers to obtain children’s location, their trajectory and speed of movements, Bluetooth-based proximity tags to examine face-to-face contacts of individuals, and multi-motion receivers (MMR) to obtain the physical activity level of children. This approach enabled the monitoring of children's activities in the schoolyard, their contacts with peers, and their movements within the environment during unstructured breaks at school. Multimodal analyses of sensor data yielded a detailed, precise picture of children’s interactions with peers and their direct environment. Data and results obtained through these new methods based on sensor data were validated using video observations of these schoolyard events. 

Second, we aimed to distinguish between three interconnected types of affordances (physical, social, and cultural) and gave each of these explicit and measurable definitions (see Box 1). By operationalizing these terms, the data could be interpreted with greater precision and less bias. To illustrate the value that these data can have, we analysed data collected from two schoolyards. Integrating the sensor data based on the triad of affordances, in addition to providing extensive information on each individual aspect, provided extensive interdisciplinary knowledge on how the physical environmental features affected children’s social participation and movements. In addition, it highlighted how the presence of other individuals affected children’s behaviour and movement in the physical space. It also revealed how the rules set by schools and supervisors affected children’s social participation and use of space.

Third, by considering the relevance of these data, we aimed to better understand how the collected data and their analyses could inform schools, designers, and policymakers about the possibilities and limitations a schoolyard presents, and plan for practical solutions and improvements, particularly with respect to the individual differences in effectivities of vulnerable children.

## 2. Methodology

Our methodology addressed two main goals simultaneously. On the one hand, we developed a setup of sensors to gather data in the schoolyards, and an approach to work with these, in the context of two schools. On the other hand, we also carried out a simultaneous study where we gathered data from children in these schoolyards and analysed them to gain insight on children’s behaviour, and their relation to the three environmental layers (physical, social and cultural). This section presents this integrated methodology.

### 2.1. Selection

We developed our data-driven approach and applied our approach in the context of two primary special-needs schools that were geographically located in the centre of the Netherlands. The schools and parents were informed about the purpose and planning of the study, and supplied written consent for the children to participate in it. Approval for the study was obtained from the Leiden University Ethical Committee. The data-management procedures were registered and approved by the Leiden University Research Data Management Plan. 

All sensor belts were prepared and handed over to the teachers in a box 15 minutes before the break. Due to the COVID-19 restrictions, the examiners were not allowed to be in the classrooms to help children with their sensors. Instead, prior to data collection, a video presentation was shown to children that explained the research in simple words, to prepare them for participating in this study and instruct them on the use of sensor belts during the break. Teachers further helped children to put on the sensors since they were fully aware of the instructions for the sensors and each child’s specific preferences or capacity. During this process or during the break, children could refuse to wear the sensor belt, if they were not comfortable with it, which occurred in 2% of the breaks among 1–2 children.

### 2.2. Reconnaissance

Prior to data collection, the researchers visited each school for a reconnaissance visit (i.e., to explore the situation with an aim to define a strategy) for investigating the physical, social, and cultural environment. This first contact gave them the opportunity to familiarise themselves with the schoolyard, explore its environmental features, and conduct informal interviews with the school director, teachers, and caretaker during a tour of the school building and its surroundings. This go-along approach is common at the exploratory stage of similar research [36,37]. Interviews included questions about school customs, teacher and pupil preferences, habits, the organisation of breaks, and schoolyard activities. In combination with the visual inspection of the schoolyard, these provided an initial impression of physical, social, and cultural affordances, and led to hypotheses about the social and cultural context of school breaks, as well as to the selection of locations where sensor facilities should be positioned. Ultimately, the reconnaissance visit informed us about: (1) the proper locations for installing the sensor equipment and for video observers, (2) general rules about breaks (e.g., that children were not allowed to stay in classrooms except on special occasions), (3) and general rules on the use of the schoolyard, such as soft boundaries and area allocation during breaks. 

### 2.3. Participants

A total of 150 children aged between 5 and 15 years old participated, from 21 different classes from two schools (schools A and B). Data collection took place over a period of two weeks at each school, respectively, split between two recess times on consecutive days for each class. Each measurement lasted between 15-50 minutes, depending on the playgroup (younger children usually have longer breaks than older children).

Many students at school A came from mainstream education, without a specific diagnosis, because they needed extra care and support and their well-being was often under pressure due to learning pace, large-size classes, or overwhelming contact with others. The school, therefore, offers more structure, predictability, personal attention, and specialist support to improve their well-being. The majority of pupils were undiagnosed, or their diagnoses were unknown to us (63%). Of the rest, most had ADHD (20%) and autism (14%) as their primary or secondary diagnosis. In total, fifteen measurements were conducted in seven days in the period of two weeks.

**Figure 1:**
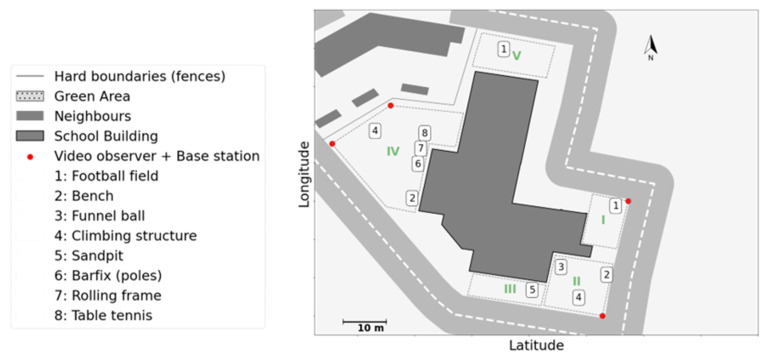
The layout of school A.

This school is located in an urban residential area where streets abutted the school on three sides and the backyards of single-family homes abutted school property on the fourth side. Hard borders (e.g., fences or walls) separated the school area from its neighbours. As shown in Figure 1, schoolyard use is separated into two parts, with junior classes being allocated a different part (sub-areas I, II, and III) from that of the senior classes (sub-areas IV and V). 

School B offers education to children whose development is disrupted or at risk of disruption due to reasons such as behavioural problems, emotional problems, or psychiatric issues. Seventy-six percent of students were autistic, and 34% with ADHD as their primary or secondary diagnosis. Therefore, these two conditions accounted for the majority of students in School B.

**Figure 2:**
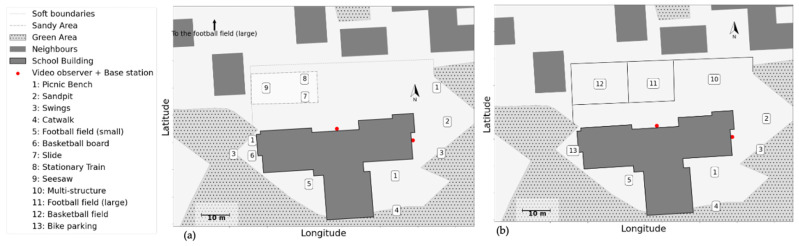
Layout of school B: (**a**) before renovation, (**b**) after renovation.

The school is located in a rural area, in close proximity to green spaces. It is located on the site of a larger complex of special-needs facilities. In the first part of our study (Figure 2a), the school shared some outdoor areas of the complex, notably a football field. On the south side, the school bordered residential properties with green areas in between. The schoolyard was therefore demarcated by soft borders on practically all sides. During our study, the schoolyard was renovated. The new layout included a harder yet penetrable separation from the complex, and a higher degree of self-sufficiency, primarily thanks to its own football field (Figure 2b). Data collection was conducted in three waves: (1) before the renovation, (2) after renovation, and (3) following minor, local improvements in the renovated schoolyard (6 months after the renovation). In total, eighteen measurements were conducted in six days in the period of two to three weeks per data collection wave.

### 2.4. Validation of Measures Obtained through Sensor Techniques

Two video recorders were present to supply validation for the data collected during the breaks using sensor techniques. Locations for video observers and sensor equipment were determined during reconnaissance visits. In the current study, video recordings were used for visually verifying the sensor data analysis and supporting the observations presented in the result section. Specifically, video recordings were used along with data analysis to ensure that the obtained results and interpretations aligned with what actually happened during the break. For this purpose, all video recordings were stamped with the date and time of the measurements per second. This enabled us to extract a particular period from the sensor data and the corresponding video recordings to verify the results obtained by the data analysis. For example, the presence of children around the ping pong table, the frequent use of the icy slide, and the popularity of the new multi-functional structure were all verified via the video recordings. 

### 2.5. Variables and Measures

**Figure 3:**
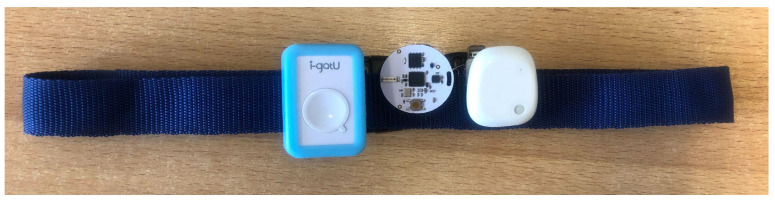
The proposed sensing system: GPS logger, proximity tag, MMR sensor (from left to right) mounted on a belt.

As shown in Figure 3, the GPS tracker, proximity tags, and MMR sensors were used to measure and analyse children’s behaviour in different aspects and their interactions with schoolyard environments. As interactions depend on the context (i.e., the physical and social setting, and user activity in both spatial and temporal dimensions), our study requires multimodal analysis to examine the highly interconnected and sophisticated layers of physical, social and cultural affordances in schoolyards. In this concept, affordances were used as means to interpret and illuminate the result of data analysis. We further identified the main variables per sensor device that were used in the identification of each affordances layer as follows:

#### 2.5.1. GPS Loggers

GPS loggers record the location of the wearer, allowing us to track the movement of each child in the schoolyard, i.e., the trajectories they follow and the places they visit. The GPS loggers used were of the i-gotU GT-120 USB type. Noise in the GPS data was removed by keeping only sequences where at least five successive points were situated within a distance of 10 metres of the schoolyard outline, to account for positional accuracy of GPS loggers. We excluded data points with unrealistic speed (10 m/s cut-off point) [38,39]. The remaining data points were used for further analysis. In schoolyard affordances, GPS locations were adopted in two directions:

- *Trajectories* of children contained the longitude and latitude of movements, through which the speed of movements was calculated (speed = displacements over time).
- *A kernel density estimate (KDE)* estimated the distribution of GPS locations in a playgroup and assessed the most visited areas.

#### 2.5.2. Proximity Tags

Proximity tags used in the research were OpenBeacon, with two base stations (Beagle-Bone Black minicomputer augmented with custom OpenBeacon hardware). Proximity tags registered each other via Bluetooth at a distance of up to 1.5 metres. They wirelessly sent data on these sightings to the base stations, which received signals 4 times per second [40,41] and registered information broadcast by tags up to 25 metres away. The proximity tags were used to detect face-to-face contacts between subjects during recess. Since most children were involved in active play, their body movements, or interfering objects, such as other individuals passing by and toys, may have interrupted the signal. To compensate for this error, the raw proximity data was interpolated by joining two successive contacts between the same peers, if the time gap between the two contacts was less than a certain threshold (35 seconds in our study) [42]. The obtained variable was defined as follows:

- *Spatial contacts* were calculated by taking the face-to-face contacts from the proximity tag and fusing it with GPS locations. This gave crucial information on where contacts took place in the schoolyard.

#### 2.5.3. MMR sensors

The MMR sensor is a wearable device that includes a BMI160 6-axis Accelerometer and Gyroscope, a BMM150 3-axis Magnetometer, and allows continuous monitoring of activities along three axes. In schoolyard affordances, MMR data is used as the following variable:

- *Spatial Activity Level* is determined based on cut-off points proposed by Puyau et al. [43], who validated accelerometer-based activity against energy expenditure (EE) in children within a 15-second time frame. We were able to adopt Puyau’s setpoints because: (1) The average participants' age was similar to our study (6–16 years old), (2) The activities performed in the validation study were the same as children’s activities in the schoolyard (walk, run, free-living activities such as computer games, playing with toys, aerobics, skipping, jump rope, soccer). This variable was date- and time-matched to each 1-second GPS data point to obtain how the activity level was related to environmental features and physical affordances.

Participants wore the three devices mounted on a belt of adjustable length, to be worn around the waist. Subjects were asked to wear the belt only during the break and to take it off when the break ended. Teachers supervising the children during the break were also issued with sensor belts to capture their contacts with pupils. As established at the reconnaissance visit, all children were required to leave the classroom during breaks, except when the weather, sickness or other major problem made it unwise.

## 3. Results

By adopting the above data-driven approach, we effectively carried out extensive data collection in two schoolyards. In this section, we present the results of our data analysis, which examined relations between children’s behaviour and environmental characteristics. All analyses were performed in Python 3.6.1, within the Anaconda environment. Geographical data for location identification was extracted from the OpenStreetMap. For all three sensors, time was used as a unique identifier (uid), and the merging of datasets was based on the recorded timestamps.

### 3.1. Physical Affordances

**Figure 4:**
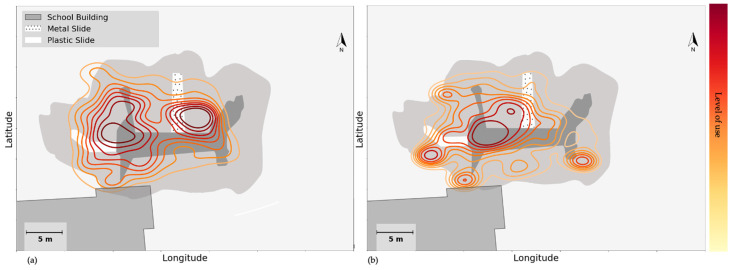
Use of space, around the slides on multi-functional structure, analysed by GPS data, during (**a**) the morning break, and (**b**) the lunch break in school B. The level of use is analysed via GPS data, and obtained contours are colour coded from the lowest level of use to the highest following the colour bar (from yellow to red).

With respect to physical affordances, the data revealed that the availability of equipment and furniture, as well as their condition, could be critical to attracting attention and activity. Figure 4 shows the KDE plot of all groups in the (a) morning break and (b) lunch break at school B, around the multi-functional structure that was one of the key new features in the renovated schoolyard. This structure includes one plastic slide (solid white colour in Figure 4) and a metal slide (white with dot hatch), as well as stairs, rope and rock climbing, a catwalk area, and a spinner structure. On that particular day, due to the cold weather and low ambient temperature, the metal slide had frozen during the morning break. Since an icy surface has lower friction, the metal slide afforded higher sliding speeds and was, therefore, a popular spot, with a higher traffic density in comparison to the plastic slide in Figure 4a. Figure 4b shows the situation at the same structure during the lunch break: by that time, the temperature had risen, rendering the metal slide less speedy and therefore less popular. In fact, at that time spatial density around the plastic slide was higher.

**Figure 5:**
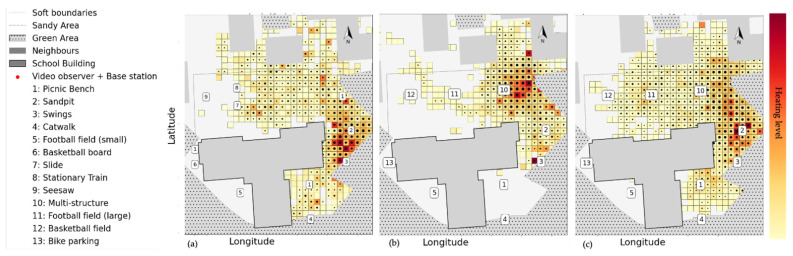
Young children’s heatmap, analysed via GPS data in school B (**a**) before the renovation, they were attracted to the newly added structure, (**b**) after the renovation, and then back to their old habitat during (**c**) the follow-up. The colour bar shows the duration of the visit. The warmer the colour (following the colour bar from yellow to red), the higher the duration of visits by children. The black dots indicate the number of children in each segment. The larger dots show a higher number of children in that spatial bin.

This sophisticated, unusual play structure did spark children’s curiosity, and became a popular spot after it was added to the schoolyard renovation. As the heatmap of young children in Figure 5a shows, before the schoolyard was renovated, the most heated spots were the sandpit and swings (Spots 2 and 3) and the areas around them where children could cycle around. Adding the new multi-structure on Spot 10 attracted children towards this new structure (Figure 5b). However, during the follow-up, many children lost interest and reverted to their old preferences (Figure 5c; on Spots 2 and 3). 

**Figure 6:**
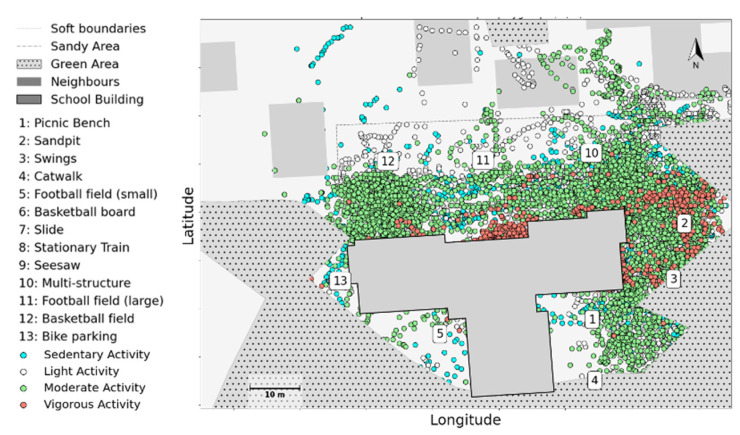
The location of physical activity levels in school B was analysed via Accelerometer data and then fused with GPS locations.

The layout of the schoolyards and the proximity of play structures relative to each other could also affect children’s movements and activities. Figure 6 shows the physical activity level of children, fused with GPS locations and then mapped to the floorplan. Having a low proportion of vigorous activities in this playgroup suggests that the schoolyard does not offer enough space for high physical activity level activities and games.

### 3.2. Social Affordances

**Figure 7:**
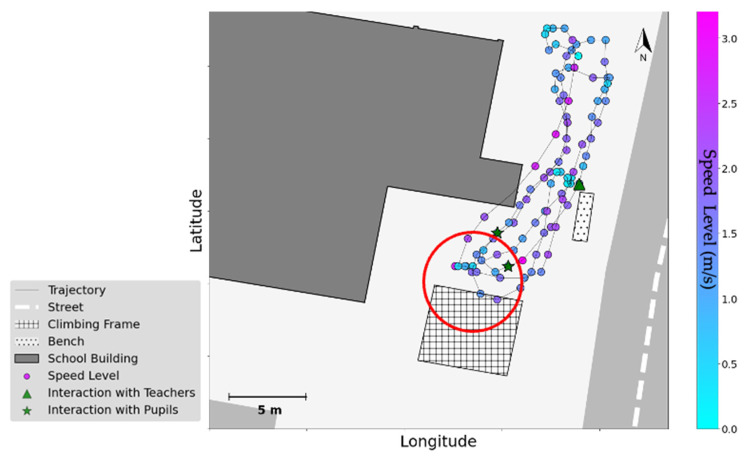
Cyclist in school A: the trajectory of movement is extracted from GPS data, and colour coded based on speed level (from light blue to light pink, following the colour bar). Face-to-face contacts are analysed from proximity tags, fused with GPS data, and mapped to the school floor plan (triangle: contacts with the teacher, and star: contacts with peers).

With respect to social affordances, it is equally clear that the density of users affects some activities such as cycling, pushing them away from crowded areas and causing intermittent trajectories. Figure 7 depicts the trajectory of a subject in school A, where the child is cycling in the schoolyard between the climbing frame and a yard fence. His speed reaches its highest value in the midway and is reduced near route endpoints and physical structures that provide opportunities for social interactions: the bench where supervising teachers are seated, and the climbing frame where peers are playing. As illustrated in Figure 7, near the climbing frame the speed not only drops to a minimum, but stationary time also increases (as shown in the red circle: data points with low-speed levels and little displacement along the trajectories around the climbing frame), suggesting the possibility of quick chats with peers. Face-to-face contacts captured by the proximity tags confirm that such social interactions occurred near the climbing frame (green stars) and at the bench (green triangle).

**Figure 8:**
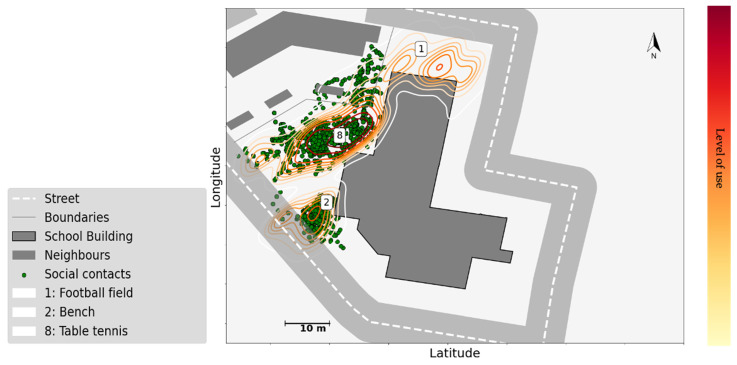
Social use of space by senior classes in school A. The level of use is analysed via GPS data, and obtained contours are colour coded from the lowest level of use to the highest following the colour bar (from yellow to red), face-to-face contacts are extracted from proximity data, fused with GPS locations and mapped to the school floor plan (note that the base station could not cover the contacts that occurred in Area 1, therefore no contacts from that region was registered).

Social affordances could also determine which physical affordances were established. Especially in senior classes, the use of space could be influenced more by who than by what. Children at this age often stay with a specific sub-group throughout the break in a certain schoolyard area that they "own". As Figure 8 shows, in school A, the areas around the tennis table and the bench were where most social contacts happened. GPS data confirm the presence of groups from senior classes in these areas. The table was used for sitting and mingling rather than for playing table tennis, which suggests that social affordances are more important than the expected use. Use of the bench by a specific group from senior classes was consistently observed across several break sessions and confirmed by the sensor data (see Figure 8).

**Figure 9:**
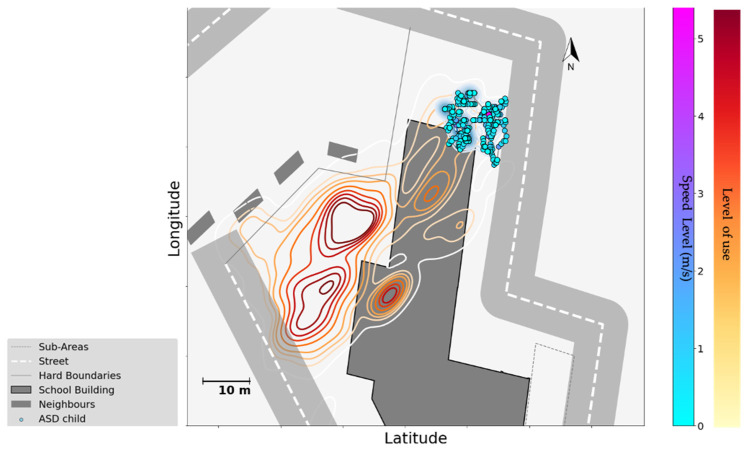
The use of space by an autistic child in comparison with its playgroup in school A. The level of use is analysed via GPS data, and obtained contours are colour coded from the lowest level of use to the highest following the colour bar (from yellow to red). The GPS location of the autistic child is plotted on the floor plan, colour coded by the speed level from the lowest speed to the highest, following the colour bar (from blue to pink).

Data also reveal movement patterns at the individual level, such as the behaviour of an autistic subject in comparison to the rest of the playgroup. Figure 9 shows the GPS data of one of the playgroups in School A, where the spatial data of an autistic subject indicates that the child remained close to the school building, avoiding dense areas.

### 3.3. Cultural Affordances 

**Figure 10:**
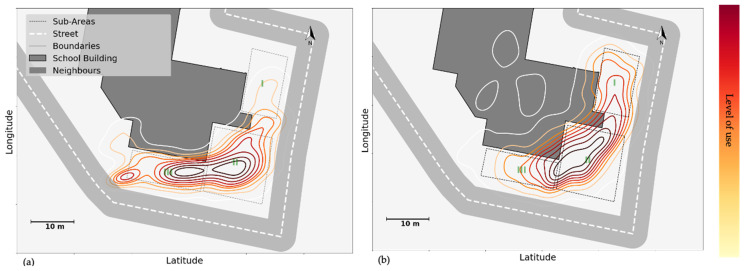
The use of space by cyclist children in school A (**a**) with restriction (**b**) without restriction from the break supervisor. The level of use is analysed via GPS data, and obtained contours are colour coded from the lowest level of use to the highest following the colour bar (from yellow to red).

Cultural affordances were quite strong, as expected for this age group and for the capacities of autistic children. For instance, in School A, younger groups were free to use all three sub-areas, I, II, and III. However, wheeled toys (e.g., bikes, steppers, scooters, etc.) were restricted to sub-area III when a specific supervisor was in charge (Figure 10a). Since biking was one of the most popular activities among young children, this restriction has a significant impact on the use of space in the schoolyard. Figure 10a shows the KDE plot when wheeled play was restricted to III: a high-density level around area III, with a peak at the end of III where the turning point was. Figure 10b shows a different day when children were not restricted to III for biking. On that day, the spatial density was widely distributed over all three sub-areas.

**Figure 11:**
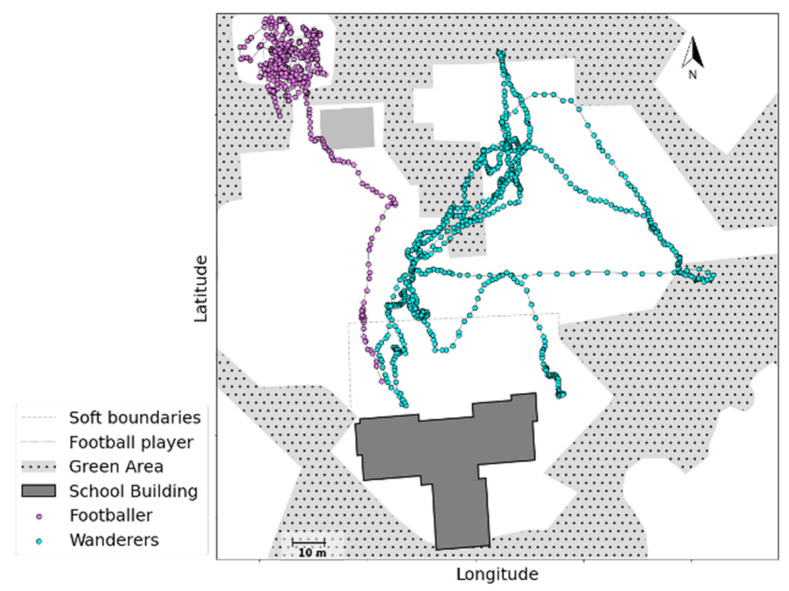
The trajectory of a group wandering around against school rules, and football players in school B, was extracted from GPS data.

Violation of school rules was only occasionally observed, for example in the trajectories of a few subjects who wandered around in School B before renovation (green dots in Figure 11). This contrasted with the trajectories of subjects who went to play on the football field: these followed the shortest route to the remote football field, with no subjects wandering off (purple dots), according to the school rules: children were not allowed to cross the soft boundaries and move around in the residential area.

Supervision naturally reinforced cultural constraints. In fact, in both schools, most constraints were designed with supervision in mind: how to make it more economical and more effective. This, however, also created an illusion of full control among the teachers, who were surprised when the researchers reported the above example: they had never observed or even suspected something like that. Otherwise, they would have taken measures to prevent it.

## 4. Discussion & Conclusions

The purpose of this study was to show a data-driven approach that examined how three environmental layers (physical, social, and cultural) interact with children’s movements and behaviour during unstructured play at recess time. Through modern sensor technologies, sensor data was collected on children’s activities in two primary special education schools in the Netherlands. The obtained data was further analysed in light of schoolyard affordances.

Our first aim was to adopt a novel sensor data-driven approach to examine affordances in schoolyards. Observations by our researchers and feedback from the school demonstrated that the belt we had designed with sensors was not distracting for children. Instead, it was exciting to primary school children, who showed eagerness to participate in the research. Moreover, our data-driven approach allowed us to register more subjects over a longer period of time cost-effectively. The sensing system in our approach includes GPS loggers, proximity tags, and MMR sensors to capture different aspects of children’s behaviour (i.e., locations, face-to-face contacts, physical activity level). Integration of this information was crucial in our study. The spatial dimensions of the face-to-face contacts and physical activities were obtained by fusing the registered data with GPS locations. This enabled us to understand how the physical characteristics of the schoolyard impact children’s contacts, physical activity levels and their use of space.

Regarding our second aim, our approach allowed us to identify three main environmental factors that influence children’s behaviours. First, the physical capacity of the schoolyard, such as its size, shape, equipment (e.g., availability and arrangement) and relevant rules and constraints, serve as preliminary triggers that affect children’s behaviours (e.g., the Sliding example in Figure 4; physical activity level in Figure 6). The schoolyard should have adequate capacity and offer a variety of options for children to play and engage in different activities. In addition, schoolyard equipment, depending on its design (e.g., climbing frames, swings, seesaw, etc.) and arrangement (e.g., materials, height, size, and proximity to other equipment) could either hinder or attract children to the equipment, and discourage or encourage play. Earlier research also confirmed that schoolyard size and availability of play equipment, such as sports facilities, recreation areas, surface materials, and greenery elements, could promote children’s physical activity level [44,45]. Green areas are also found to be a contributing factor in promoting children’s resilience and reducing their stress levels [46]. Similarly, close proximity between play structures generates more spots for physical activity [36,47]. Yet, our data showed that close proximity between different play equipment in a small space could also result in lower physical activity levels. The overall shape of the schoolyard influences the supervision method and could result in demarcations that reduce the space available to children (e.g., restricted cyclists in Figure 10, versus wandering cyclists in Figure 11). Importantly, our three-wave data collection in school B shows that new, fancy equipment may not always remain attractive after the novelty wears off. With time, children may still return to equipment that affords a wider variety of creative activities. This again emphasises the importance of examining the capacity of the schoolyard according to its affordances.

Second, opportunities for social interaction often attract children towards certain spots and motivate children to use the space for social purposes. Our data confirmed that the spots where such opportunities were offered could indeed stimulate social interaction, even when they were not originally designed for that purpose (e.g., the bench and climbing structure in Figure 7; the ping pong table in Figure 8). This outcome echoes previous findings about the impact of environmental aspects, such that green and natural elements, multi-functional equipment with diverse structures like sand-pits and stairs, (semi-)secluded places, etc., encourage children’s social interactions [48,49,50,51,52]. These features usually afford more variety and flexibility in children’s play, and may be helpful for the initiation of social activities, or offer a space to play and hang out without interruption or to recover from active play [48,52]. Conversely, popular areas and equipment can also cause more conflicts over available resources. For example, play structures such as swings, slides, seesaws, etc. can be locations that support constructive interactions, as well as foci of competition, irritation, or even bullying. 

Third, individual needs could lead to quite different patterns in the use of space. Despite the patterns shown in the above two points, our data showed that vulnerable children (e.g., autistic and ADHD children who have different effectivities), who have different capacities and needs in their interactions with the environment, may use schoolyard affordances of any kind in a unique way. This is observed via sensor data in their trajectory of movements, use of space, and activities during the break, as with the autistic child who remained next to the school building in Figure 9, away from the area where most of his/her classmates were playing. With this data-driven approach, we show that this autistic child was not just alone, but alone in a context: in the corner, with limited movements, throughout the break. Such information that links individual patterns to environmental factors, in children’s natural setting, is crucial for studying children’s behaviour, especially for understanding the needs of vulnerable children. Yet, while our sensing system enabled us to detect these differences, it is also important to identify whether these differences reflect the preferences of the child, difficulties in joining others, or social exclusion.

Our third aim was to inform schools, designers, and policymakers about these identified factors. School organisations and supervisors in schoolyards play a key role in identifying situations where children are overwhelmed or triggered by peers or certain equipment, and avert these scenarios by taking the required precautions, and overruling the existing climate [53], for example by planning a timetable for using a popular play structure by different sub-groups of children. School organisations should also deliver extra support, customised rules, and structures suited to vulnerable children, and an inclusive school climate that values diversity and individual differences. For example, the schoolyard could have different sub-areas in different colours and play structures, and children could choose in which colour (or sub-area) they would like to play before starting the break. In this way, supervisors could estimate high-demand areas and, by re-organizing the available resources, try to strike a balance in the use of space. This could deliver substantial benefits for example to children who are overwhelmed by crowded or noisy situations, such as children with hearing aids or autistic children. These findings have important implications for developing interventions that adapt the environment to promote social participation. They also show the strengths of our proposed sensing system in evaluating the intervention and providing relevant insights to schools, designers, and policymakers. 

Overall, the obtained results showed that making affordances explicit and measurable, through the use of approaches such as those presented in this paper, can help to better understand children’s behaviours and moves, translate these insights into actionable advice for schools, designers and policy makers and further improve current layouts and forms of organisation, especially with respect to the capacity of vulnerable children.

### 4.1. Limitations and Future Directions

Our proposed sensing system allowed us to register more subjects and activities with higher precision than we could have with observation methods. Yet, while a belt with sensors may be exciting to primary school children, it may not be suitable for adolescents in high school. Besides, regarding the performance of individual sensors, GPS technology showed great promise in obtaining individual positions. Yet, given GPS accuracy (1–10 m), ultra-wideband (UWB) (accuracy of 10 cm) are better suited to the scale and context of schoolyards [54,55,56]. Such accurate positioning systems could be used in identifying contacts between subjects in a more comprehensive range of actions and interactions (e.g., parallel play) than proximity tags which record only face-to-face contacts [57]. This enhancement could withdraw the proximity tags from the sensing system. In addition, in our current system, the value of contacts (e.g., does a child who is identified alone on the playground feel lonely or happy to be left alone) and moves remain unknown. For example, while we could truly obtain from sensor data that a child played alone in the sand-pit, we did not know the reason i.e., their emotions or preferences. Therefore, the sensing system could be improved in future in three ways: first, it should be made suitable for a wider age range; second, it should include a more accurate positioning system e.g., UWB; and third, it should incorporate a strategy to let participants actively express their emotions and preferences through focus group interviews and/or by providing real-time responses for example via smartwatches.

Another limitation of this study is that the obtained results were based on a small number of measurements at two special education schools. To design an automated monitoring system whose conclusions could be generalised to different environments and scenarios, more data collection would be needed. This was not possible due to the COVID-19 crisis, but currently remains one of the major foci of this research team.

Finally, our proposed data-driven approach makes it possible to analyse movement, social behaviour and environmental interactions among children at a specific school. While it makes it possible to gain a better understanding of the current challenges children face, it also holds the potential to reach beyond this understanding alone, and empower schoolyard designers to define and monitor the effect of incremental improvements to schoolyards, for example in the form of new equipment or changes in the physical organisation of the schoolyard. This approach could even be used to examine real-time adaptations to the rules and parameters of digital-physical interactive schoolyards. As such, the work presented here is the first step in what could become a long-term research program of a much wider scope.

For future studies that aim to adopt a similar approach, one of the most important lessons is that each case should be studied before collecting the data: all environmental layers (physical, social, cultural and their interrelations) must be considered in order to form expectations for the data and their analyses. As described in Section 2.2, a reconnaissance visit that featured informal interviews and inspections helped us understand what took place in each schoolyard, its specific circumstances, and the intentions of the school (which should be respected but also critically analysed). As the data analyses demonstrated, there were many factors behind observed behaviours and patterns that may go unnoticed, if researchers focus on a single aspect or goal. In other words, context matters. Depending on the research questions at stake, variables for capturing the environmental layers require clear definitions, and the particular sensing technologies should be chosen accordingly. For example, the positioning technology should be chosen depending on environmental conditions to appropriately record the location of the users, and thus reliably analyse the child’s interaction with the environment. For example, GPS technology is more suitable for large outdoor areas, while UWB is a better option for relatively small areas. Integrating background knowledge obtained during reconnaissance visits and the collected data from sensors allows for a better interpretation of environmental factors that affect children’s movements and behaviour.

## Data Availability

The data presented in this study are openly available in DANS EASY at DOI:10.17026/dans-2b6-y27b.
