# Correction: Nasri et al. A Novel Data-Driven Approach to Examine Children’s Movements and Social Behaviour in Schoolyard Environments. *Children* 2022, *9*, 1177

**DOI:** 10.3390/children9121882

**Published:** 2022-12-01

**Authors:** Maedeh Nasri, Yung-Ting Tsou, Alexander Koutamanis, Mitra Baratchi, Sarah Giest, Dennis Reidsma, Carolien Rieffe

**Affiliations:** 1 Unit of Developmental and Educational Psychology, Institute of Psychology, Leiden University, 2300 RB Leiden, The Netherlands; 2 Leiden-Delft-Erasmus Centre for BOLD Cities, Leiden University, 2300 RA Leiden, The Netherlands; 3 Department of Computer Science, Faculty of Science, Vrije Universiteit Amsterdam, 1081 HV Amsterdam, The Netherlands; 4 Faculty of Architecture & the Built Environment, Delft University of Technology, 2628 BL Delft, The Netherlands; 5 Leiden Institute of Advanced Computer Science, Leiden University, 2300 RA Leiden, The Netherlands; 6 Institute of Public Administration, Faculty of Governance and Global Affairs, Leiden University, 2511 DC The Hague, The Netherlands; 7 Human Media Interaction Research Group, Faculty of Electrical Engineering, Mathematics and Computer Science, University of Twente, 7522 NB Enschede, The Netherlands; 8 Department of Psychology and Human Development, Institute of Education, University College London, London WC1H 0AA, UK

The authors request the following corrections because the changes made according to the second round of the review process were not included in the original publication [1]. The authors state that the scientific conclusions are unaffected. This correction was approved by the academic editor. The original publication has also been updated. Below, the authors explain the corrections, and include the corrected content in-between the “**” marks. 

## Additional Affiliations

In the original publication, the affiliations of Maedeh Nasri, Yung-Ting Tsou, Alexander Koutamanis and Dennis Reidsma were not updated. The corrected affiliations are as follows: 

**

Maedeh Nasri ^1,2,^*, Yung-Ting Tsou ^1,3^, Alexander Koutamanis ^4^, Mitra Baratchi ^5^, Sarah Giest ^6^, Dennis Reidsma ^7^ and Carolien Rieffe ^1,7,8^

^1^  Unit of Developmental and Educational Psychology, Institute of Psychology, Leiden University, 2300 RB Leiden, The Netherlands
^2^  Leiden-Delft-Erasmus Centre for BOLD Cities, Leiden University, 2300 RA Leiden, The Netherlands
^3^  Department of Computer Science, Faculty of Science, Vrije Universiteit Amsterdam, 1081 HV Amsterdam, The Netherlands
^4^  Faculty of Architecture & the Built Environment, Delft University of Technology, 2628 BL Delft, The Netherlands
^5^  Leiden Institute of Advanced Computer Science, Leiden University, 2300 RA Leiden, The Netherlands
^6^  Institute of Public Administration, Faculty of Governance and Global Affairs, Leiden University, 2511 DC The Hague, The Netherlands
^7^  Human Media Interaction Research Group, Faculty of Electrical Engineering, Mathematics and Computer Science, University of Twente, 7522 NB Enschede, The Netherlands
^8^  Department of Psychology and Human Development, Institute of Education, University College London, London WC1H 0AA, UK

**

## Errors in Citation

In the original publication, the numbering of the in-text citations in the Introduction was not updated. 

A correction has been made to the 2nd paragraph of Section 1.1. (Affordances and effectivities in physical, social, and cultural environments in schoolyards):

**Physical affordances are mostly studied in children-oriented research, including studies on how different environments (e.g., home, school, sport, leisure, neighbourhood, outdoor play) either promote or hinder various motor activities [11–13], and how physical, social, and cultural affordances influence physical activity levels in schoolyards [14,15].** 

A correction has been made to the 4th paragraph of Section 1.1. (Affordances and effectivities in physical, social, and cultural environments in schoolyards):

**For example, autistic children may be sensitive to certain ambient triggers (sounds, light, or touch) or avoid being in crowded areas [16–18]. They often prefer repetitive games with predictable results, such as spinning, twirling, and illuminating [19,20], and fixed routines, with clear instructions and rules to follow [21]. Autistic children can also find initiating or maintaining social contacts with other peers quite challenging [22]. Children with attention deficit hyperactivity disorder (ADHD) are observed to often change activities during breaktime, and many ADHD children have difficulties sustaining interactions with peers [23–25]. Thus, for vulnerable children, it may be especially critical to unravel the relationship between their individual interactions and their environment.**

A correction has been made to the 6th paragraph of Section 1.1. (Affordances and effectivities in physical, social, and cultural environments in schoolyards):

**To the best of our knowledge, there are no prior studies that examine how the environmental triad of physical, social, and cultural affordances interact with children’s behaviours and movements in the schoolyard. Moreover, in available affordance studies, vulnerable children have never been considered. While a large body of literature has reported on vulnerable children’s physical activity levels, forms of play, and social connectedness in the schoolyard [26–29], outcomes that were reported were not linked to any environmental factors.**

A correction has been made to Box 1. (Affordances in schoolyards), in the description of “Physical affordances”:

**Physical affordances: what the physical layout and features of the schoolyard afford to children and their activities. These are critical for many vulnerable children, to the extent that they may even exclude themselves from what takes place in the schoolyard. For example, what most humans tolerate as mild background noise can be insufferable to children with cochlear implants, who, consequently, tend to refrain from entering schoolyard areas where exposed to such noise [30,31].**

A correction has been made to the 1st paragraph of Section 1.2. (Present study):

**This new data-driven approach has been used, for example, to examine physical activity levels [32], and to compare active outdoor play in schoolyards and in natural environments, taking into account personal characteristics (e.g., age) as well as the physical and social environment [33–36].**

A correction has been made to the 3rd paragraph of Section 4. (Discussion & Conclusions):

**Green areas are also found to be a contributing factor in promoting children’s resilience and reducing their stress levels [46]. Similarly, close proximity between play structures generates more spots for physical activity [36,47].**

## Error in Figure

In the original publication, there was a mistake in the numbering of figures. Corrections have been made to the figure numbers:

In the 3rd paragraph of Section 4. (Discussion & Conclusions): 

**Regarding our second aim, our approach allowed us to identify three main environmental factors that influence children’s behaviours. First, the physical capacity of the schoolyard, such as its size, shape, equipment (e.g., availability and arrangement) and relevant rules and constraints, serve as preliminary triggers that affect children’s behaviours (e.g., the Sliding example in Figure 4; physical activity level in Figure 6). The schoolyard should have adequate capacity and offer a variety of options for children to play and engage in different activities. In addition, schoolyard equipment, depending on its design (e.g., climbing frames, swings, seesaw, etc.) and arrangement (e.g., materials, height, size, and proximity to other equipment) could either hinder or attract children to the equipment, and discourage or encourage play. Earlier research also confirmed that schoolyard size and availability of play equipment, such as sports facilities, recreation areas, surface materials, and greenery elements, could promote children’s physical activity level [44,45]. Green areas are also found to be a contributing factor in promoting children’s resilience and reducing their stress levels [46]. Similarly, close proximity between play structures generates more spots for physical activity [36,47]. Yet, our data showed that close proximity between different play equipment in a small space could also result in lower physical activity levels. The overall shape of the schoolyard influences the supervision method and could result in demarcations that reduce the space available to children (e.g., restricted cyclists in Figure 10, versus wandering cyclists in Figure 11). Importantly, our three-wave data collection in school B shows that new, fancy equipment may not always remain attractive after the novelty wears off. With time, children may still return to equipment that affords a wider variety of creative activities. This again emphasises the importance of examining the capacity of the schoolyard according to its affordances.**

In the 4th paragraph of Section 4. (Discussion & Conclusions): 

**Our data confirmed that the spots where such opportunities were offered could indeed stimulate social interaction, even when they were not originally designed for that purpose (e.g., the bench and climbing structure in Figure 7; the tennis table in Figure 8).** 

In the 5th paragraphs of Section 4. (Discussion & Conclusions): 

**This is observed via sensor data in their trajectory of movements, use of space, and activities during the break, as with the autistic child who remained next to the school building in Figure 9, away from the area where most of his/her classmates were playing.**

## Text Correction

There are several grammar errors in the original publication, which should be corrected as follows:

The use of prepositions has been corrected in:

The first sentence of the Abstract:

**Social participation in schoolyards is crucial for children’s development.**

The 2nd paragraph of Section 3.3. (Cultural affordances):

**Violation of school rules was only occasionally observed, for example in the trajectories of a few subjects who wandered around in School B before renovation (green dots in Figure 11). This contrasted with the trajectories of subjects who went to play on the football field: these followed the shortest route to the remote football field, with no subjects wandering off (purple dots), according to the school rules: children were not allowed to cross the soft boundaries and move around in the residential area.**

The 1st paragraph of Section 4.1. (Limitations and future directions):

**This enhancement could withdraw the proximity tags from the sensing system. In addition, in our current system, the value of contacts (e.g., does a child who is identified alone on the playground feel lonely or happy to be left alone) and moves remain unknown. For example, while we could truly obtain from sensor data that a child played alone in the sand-pit, we did not know the reason i.e., their emotions or preferences.**

In the original publication, there was a redundant “e.g.” in the text. A correction has been made to the 2nd paragraph of Section 1.1. (Affordances and effectivities in physical, social, and cultural environments in schoolyards):

**Heft distinguished between ten types of outdoor environments, such as “flat, relatively smooth surfaces” (which may afford walking, running, cycling, skating, skateboarding) or “attached objects” (which may afford sitting-on, jumping-on/over/down-from), and further extends affordances to include social and emotional behaviours [9,10].**

In the original publication, a comma was missing after “However” and “For example”. A correction has been made to item b. of Box 1. (Affordances in schoolyards):

**How the presence of others adds to or detracts from the affordances of the physical environment. For example, if a swing is already occupied by another person, then the child is unable to sit on it. However, a new affordance becomes available: for example, pushing the person sitting on the swing.**

In the original publication, there was “and” instead of comma in the text: 

A correction has been made to the 3rd paragraph of Section 1.2. (Present study):

**This technology included GPS loggers to obtain children’s location, their trajectory and speed of movements, Bluetooth-based proximity tags to examine face-to-face contacts of individuals, and multi-motion receivers (MMR) to obtain the physical activity level of children.**

A correction has been made to the 3rd paragraph of Section 4.1. (Limitations and future directions):

**Finally, our proposed data-driven approach makes it possible to analyse movement, social behaviour and environmental interactions among children at a specific school.**

In the original publication, a comma was missing after “i.e.” and the word “cultural” was missing in the 1st paragraph of Section 2.2. (Reconnaissance). The corrected text is as below:

**Prior to data collection, the researchers visited each school for a reconnaissance visit (i.e., to explore the situation with an aim to define a strategy) for investigating the physical, social, and cultural environment.**

In the original publication, present-tense verbs were sometimes used in the Methods section. A correction has been made to the 2nd paragraph of Section 2.3. (Participants):

**Many students at school A came from mainstream education, without a specific diagnosis, because they needed extra care and support and their well-being was often under pressure due to learning pace, large-size classes, or overwhelming contact with others. The school, therefore, offers more structure, predictability, personal attention, and specialist support to improve their well-being. The majority of pupils were undiagnosed, or their diagnoses were unknown to us (63%).**

In the original publication, the use of the preposition before “schoolyard” was incorrect, and the word “Section” was missing when Section 2.2 was being referenced to. A correction has been made to the 4th paragraph of Section 4.1. (Limitations and future directions):

**As described in Section 2.2., a reconnaissance visit that featured informal interviews and inspections helped us understand what took place in each schoolyard, its specific circumstances, and the intentions of the school (which should be respected but also critically analysed).**

In the original publication, the information about the approved ethics applications was not updated. A correction has been made to the “**Institutional Review Board Statement**” and the “**Informed Consent Statement**” to include the most updated information: 

**Institutional Review Board Statement: The study was conducted in accordance with the Declaration of Helsinki and approved by the Psychology Research Ethics Committee of Leiden University (V2-2428—date 04 June 2020; V3-2685—date 24 October 2020). The data management procedures were registered and approved by the Leiden University Research Data Management Plan.**

**Informed Consent Statement: Informed consent was obtained from all children’s parents involved in the study before the test procedures. The informed consent form and the research protocol were approved by the Psychology Research Ethics Committee of Leiden University (V2-2428-date 4 June 2020; V3-2685—date 24 October 2020).**

There was an error in the original publication in the “**Conflicts of Interest**” statement. “No conflicts of interest were reported” should be added as the starting sentence. A correction has been made to this statement: 

**Conflicts of Interest: No conflicts of interest were reported. This paper represents independent research part-funded by the Dutch Research Council (NWO) and Leiden-Delft-Erasmus Centre for BOLD Cities.**
